# Betul-Ol Versus Salicylate of Methyl

**Published:** 1904-04

**Authors:** Gustave Foullon

**Affiliations:** New York


### Betul-ol versus Salicylate of Methyl.

By GUSTAVE FOULLON, New York.

WHILE it would be absurd to contest the therapeutic value of the synthetical salicylate of methyl, yet it cannot be denied that actual experiment with the natural product derived from the "betula lenta" gives much better physiologic results, although even this may cause slight irritation of the skin.[1] Synthetic methyl salicylate has a variable specific gravity (1.183 to 1.190), whereas methyl salicylate as true betula lenta oil, is 1.180 at 15° C. The artificial product of commerce always contains toxic homologous esters, owing to the presence of impurities in the salicylic acid from which it is made (which in turn is derived synthetically from carbolic acid), and while it may not be actually impossible to purify such commercial methyl salicylate of these poisonous constituents, the cost of so doing, would be much greater than the harmless product of the sweet birch (*betula lenta*).

Natural methyl salicylate occurs in the volatile oils of wintergreen and sweet birch, both of which differ in certain respects from the synthetic product obtained by the condensation of methyl alcohol and salicylic acid. True oil of wintergreen contains about 90 per cent. of the methyl ester of salicylic acid, together with small quantities of a paraffin (m. p. 65.5° C.), an aldehyde, an alcohol, and an ester which, upon saponification, breaks up into the same alcohol and an acid. The oil from *betula lenta* is more than 99 per cent. of methyl salicylate, together with traces of the same paraffin and an ester. It is noteworthy that methyl salicylate does not preëxist as such in the plant, but is liberated by the action of a ferment (betulase) upon a glucoside (gaultherin),

1. Vidal. *Pharm. Journ.*, London, 1897, p. 459.

the result of the hydrolysis which takes place being represented as follows:

$$C_{14}H_{18}O_{8}+H_2O=C_6H_{12}O_6+C_6H_4\left[\begin{matrix}OH\\COOCH_3\end{matrix}\right.$$

Dr. Van Romburgh of the botanical garden at Buitenzorg, Java, has recently furnished additional proof of the wide distribution of methyl salicylate in the vegetable world. Of 900 various plants examined for volatile constituents, 160 or 18 per cent. rendered a distillate containing more or less of this ester. The absorption of the salicylate of methyl by the skin, was first indicated by the French physicians Lannois and Linossier. The ester is saponified in the blood, and the salicylic acid is eliminated in the urine in the form of sodium salicylate.

Betul-ol is a compound methyl-oleo-salicylate containing menthol. It has many advantages over the artificial and natural methyl salicylate in treating the symptom pain arising from rheumatism, neuralgia, sciatica or in fact from any cause. It is very rapidly absorbed, affording almost instant relief without reddening the skin or producing irritation. Its anodyne and antiseptic action takes place, moreover, even if the betul-ol is not applied over the seat of the pain, because this salicylic compound, when taken up through the circulation, on coming in contact with any lesion, finds itself in the presence of an excess of carbonic acid which liberates salicylic acid at the spot. Hence direct antiseptic results are produced in the tissues, followed by relief from pain. Effete cell tissue and pus are sterilised by the free salicylic acid and its application is followed by slight diuresis and a marked increase of all solid constituents of the urine, especially urea and uric acid. Thus we understand why the salicylates are *antiseptic, eliminative, cholagoguic, antithermic* and *analgesic,* and so useful in rheumatism (acute and chronic), rheumatoid arthritis, muscular rheumatism, lumbago, neuralgia, sciatica, and even in gout and the fulgurating pains of locomotor ataxia.

The counterirritant, penetrating power and local anesthetic properties of betul-ol are indicated in all arthritic, neuralgic and myalgic affections, including arthritis from any cause, but particularly those affecting the superficial nerves, and any form of myalgias, which have always been counted among the most stubborn affections. It has been applied in the treatment of endometritis and its complications, salpingitis, etc., and especially in gonorrheal cervical endometritis (apply by painting the cervix); it also relieves pruritus and lichen simple. Betul-ol in fact is applicable wherever we look for local antirheumatic, antipodagric and antiseptic results. Betul-ol yields on analysis, the same percentage of salicylic acid as salicylate of soda; it contains no morphine or cocaine, but these may be added in prescribing if desired; it is soluble in all proportions of ether, alcohol, chloroform or oils, and may be used either pure or in combination as a liniment.

Betul-ol is for *external* use only; but colchi-sal capsules *internally,* are essential to the treatment, *where there is a mixed rheumatic and gouty tendency.* Quite small amounts of betul-ol suffice to give relief, as each minim absorbed produces an equivalent of one grain of nascent salicylic acid. To facilitate the absorption of betul-ol, the affected surface should first be washed with soap and water to dispose of the excess of fat normally found in the skin. Apply with the camel-hair brush if the affected parts be very tender; gentle friction, however, is recommended where it can be borne. To insure proper absorption after application, the part should be covered with a soft impermeable tissue (gelatino-silk tissue is the best) and afterward enveloped in cotton wool to maintain the temperature.

